# Foetal oestrogens and autism

**DOI:** 10.1038/s41380-019-0454-9

**Published:** 2019-07-29

**Authors:** Simon Baron-Cohen, Alexandros Tsompanidis, Bonnie Auyeung, Bent Nørgaard-Pedersen, David M. Hougaard, Morsi Abdallah, Arieh Cohen, Alexa Pohl

**Affiliations:** 1 Autism Research Centre, Department of Psychiatry, University of Cambridge, Cambridge, UK; 2 Department of Psychology, School of Philosophy, Psychology and Language Sciences, University of Edinburgh, Edinburgh, UK; 3 Department for Congenital Disorders, Danish Center for Neonatal Screening, Statens Serum Institute, Copenhagen, Denmark; 4 Department of Child and Adolescent Mental Health Services, Region of Southern Denmark, Hospital of Southern Jutland, Aabenraa, Denmark

**Keywords:** Predictive markers, Diagnostic markers

## Abstract

Elevated latent prenatal steroidogenic activity has been found in the amniotic fluid of autistic boys, based on measuring prenatal androgens and other steroid hormones. To date, it is unclear if other prenatal steroids also contribute to autism likelihood. Prenatal oestrogens need to be investigated, as they play a key role in synaptogenesis and corticogenesis during prenatal development, in both males and females. Here we test whether levels of prenatal oestriol, oestradiol, oestrone and oestrone sulphate in amniotic fluid are associated with autism, in the same Danish Historic Birth Cohort, in which prenatal androgens were measured, using univariate logistic regression (*n* *=* 98 cases, *n* *=* 177 controls). We also make a like-to-like comparison between the prenatal oestrogens and androgens. Oestradiol, oestrone, oestriol and progesterone each related to autism in univariate analyses after correction with false discovery rate. A comparison of standardised odds ratios showed that oestradiol, oestrone and progesterone had the largest effects on autism likelihood. These results for the first time show that prenatal oestrogens contribute to autism likelihood, extending the finding of elevated prenatal steroidogenic activity in autism. This likely affects sexual differentiation, brain development and function.

## Introduction

The male-biased prevalence of autism [1, 2], together with the finding that autistic girls have a higher mutational load than autistic boys [3–5], suggests that males have a higher likelihood of developing autism. The sex ratio in autism diagnoses persists even after taking into account under- and/or mis-diagnosis, as well as camouflaging in females, with males being three times more likely to have the condition [6]. This implicates mechanisms of sexual differentiation in the development of autism. Five recent findings support this inference.

First, autistic women have atypical brain structure in sexually dimorphic regions, when assessed via magnetic resonance imaging and compared to neurotypical controls [7]. Second, functional connectivity in the brain of males with autism shows both hypermasculine and hyperfeminine patterns, when assessed in relation to neurotypical sex differences [8]. Third, autistic people show a masculinised shift in scores on two key sexually dimorphic psychological traits, empathy and systemising, a finding that has been replicated in a big data study of 36,000 autistic people [9, 10]. Fourth, autistic women have elevated androstenedione levels, the precursor to testosterone [11]. Finally, fifth, autistic children have hypermasculine facial features, as rated using three-dimensional photogrammetry [12].

Although autism is strongly heritable and sex-associated genetic mechanisms could contribute to this implication of sexual differentiation in autism [2, 5], prenatal hormone exposure and a brief surge in foetal testosterone are critical for sexual differentiation and masculinisation in humans [13, 14]. In line with this, we previously found elevated steroidogenic activity during this prenatal masculinisation window (PMW) in the amniotic fluid of autistic boys [15]. Subsequently, three very large epidemiological studies revealed a link between autism and maternal polycystic ovarian syndrome (PCOS), a condition associated with androgenic excess [16–18]. Consistently with this, the 2D:4D digit ratio, a marker of prenatal androgen exposure, is also masculinised in autistic children and their parents [19]. Finally, autistic women and their mothers have elevated rates of steroid-related cancers, such as breast cancer and ovarian cancer [20].

However, a number of studies that focused on testosterone have not replicated the correlation of hormonal levels with autistic traits. First, umbilical cord testosterone measured soon after birth was not associated with the development of autistic traits [21]. Second, salivary testosterone during a brief period of postnatal steroid surge (‘mini-puberty’) also did not correlate with autistic traits in toddlers [22]. In both cases, testosterone was measured postnatally—in the neonatal period—rather than during the PMW, during which foetal testosterone is first produced and masculinisation of the brain and body commences. This would suggest that timing is critical for the effects of testosterone on the brain, with the late first-early second trimester PMW being key, rather than the neonatal period. Finally, univariate assessment of amniotic testosterone in a separate cohort of neurotypical children also failed to reveal an association to autistic traits in childhood [23]. This latter finding may reflect that the wider endocrine environment outside testosterone is also significant for autism likelihood.

While prenatal androgens are responsible for masculinisation in humans, prenatal oestrogens also contribute to foetal and neonatal brain development [24], and yet these have not been thoroughly investigated for their potential role in autism likelihood. Oestrogens and their receptors are widespread in the developing brain in both males and females and regulate many neurodevelopmental processes, including synaptogenesis, apoptosis and neuronal differentiation [25–27]. Oestradiol in particular supports synapse formation in the cortex by enhancing excitatory GABA activity [28]. In autism, synapse formation [29], neuronal differentiation [30] as well as the GABAergic system [31] are all atypical. These provide clues that prenatal oestrogens may be involved in autism. However, we still lack direct evidence of this.

With regard to clinical studies in humans, low oestriol in maternal serum during the second trimester of pregnancy significantly increases the likelihood of autism in the foetus, as demonstrated in a large study of *n* = 2586 autistic pregnancies [32]. This study may have been confounded by a variety of pregnancy complications, such as pre-eclampsia [33] and being small for gestational age [34], since these are also more frequent in autism [35–37]. Thus, further study of prenatal oestrogenic activity, particularly in foetal circulation, is warranted. In addition, there is a need to compare different prenatal oestrogens to each other, in relation to autism likelihood.

In the present study, we measured prenatal levels of oestriol, oestradiol, oestrone and oestrone sulphate in amniotic fluid of boys with and without autism (*n* = 98 and *n* = 177 respectively) from the Danish Historic Birth Cohort (HBC), in the same samples in which we had found an elevated steroidogenic factor, following principal component analysis of prenatal androgens and other steroid hormones [15]. We have expanded on these findings by assaying oestrogens and by assessments of each steroid hormone to autism likelihood via univariate logistic regression. We also investigated potential differences in the aromatising capacity in autism by comparing the ratio between androgens and oestrogens. Finally, we calculated standardised effect sizes for all hormones assayed to date in this cohort, in order to understand which amniotic fluid hormones make the largest contribution to autism likelihood.

## Methods

### Participants and laboratory methods

The study was approved by the Danish Data Protection Agency and The Danish Ethical Committee of Midtjylland Region. The Danish Historic Birth Cohort was established at Statens Serum Institute, Copenhagen with a grant from The Danish Medical Research Foundation and The Danish Ministry of the Interior and Health (Project no 271-05-0523/09-060179). A full description of the cohort selection procedure is available elsewhere [15]. Briefly, cases and controls were drawn from singleton births between the years 1993 and 1999 inclusive, whose amniotic fluid samples were stored in the HBC. These corresponded to amniocentesis procedures performed between 14 and 16 weeks of gestational age. Cases were identified from the Danish Psychiatric Central Register using ICD-10 autism spectrum codes F84.0 (childhood autism), F84.5 (Asperger’s syndrome), F84.1 (atypical autism), F84.8 (other pervasive developmental disorder) and F84.9 (unspecified pervasive developmental disorder). Any additional amniotic fluid was assayed for oestradiol, oestriol, oestrone, and oestrone sulphate, using liquid chromatography-tandem mass spectroscopy (Supplementary Table 1). As some individuals did not have sufficient remaining sample for analysis, the sample size was slightly decreased. The same data quality screening criteria that were used in the initial analysis were applied: i.e. removal of outliers >99%, removal of records where duplicate assay values were >3 SD (standard deviation) apart [15]. After this step, the final sample with high-quality data for all steroids assayed to date consisted of 98 males with autism and 177 control males. This sample was used for all analyses in this paper, unless otherwise specified (Supplementary Fig. 1).

### Statistical analyses

We calculated a correlation matrix for all assayed steroid hormones using Pearson’s correlation coefficient. We also examined the univariate distribution of each of the oestrogens. All hormones showed a substantial rightward skew. We transformed the oestrogens using the Box–Cox procedure to reduce their skew, as the distribution of the predictor variable affects the statistical power of logistic regression [38]. We used univariate logistic regression models to determine whether each hormone separately increased autism likelihood in this cohort. As the oestrogens considered here vary in their equilibrium association constants to the oestrogen receptors, and therefore their potency [39, 40], we hypothesised that if a relationship between amniotic oestrogens and autism was found, oestradiol would have the largest effect size as the most potent oestrogen. Therefore, we also considered each hormone separately, with the goal of establishing accurate effect sizes to enable a comparison between them, and we corrected for multiple comparisons using the Benjamini–Hochberg false discovery rate (FDR). To estimate and compare the aromatising capacity between cases and controls, we calculated the aromatisation ratios, according to previously published recommendations [41], by log-transforming the raw concentration values and subsequently subtracting them according to the following formula:

Ratio 1 = log(testosterone concentration in nmol/l)−log(oestradiol concentration in nmol/l)

Ratio 2 = log(androstenedione concentration in nmol/l)−log(oestrone concentration in nmol/l)

We subsequently used nonparametric tests to compare these ratios to each other (Spearman’s rank correlation coefficient) and between cases and controls (Wilcoxon rank-sum test).

## Results

**Fig. 1:**
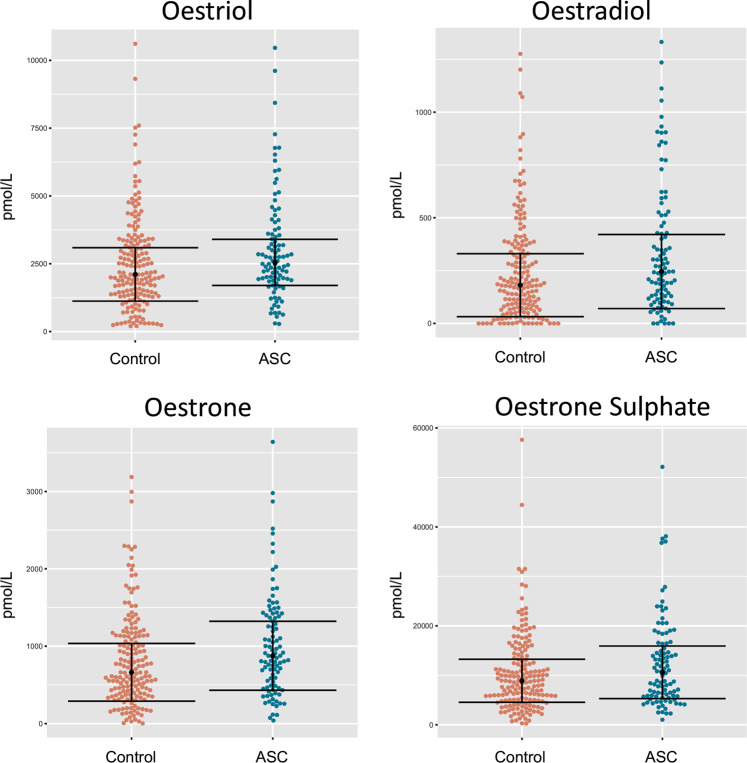
Beeswarm plots illustrating the distribution of oestriol, oestradiol, oestrone and oestrone sulphate concentrations. Error bars represent the interquartile range, and the black dot represents the median; (*n* CTR = 177, *n* ASC = 98)

There were no significant differences between groups in maternal age at birth, paternal age at birth, birth weight, gestational week at amniocentesis or storage time between groups (Table 1). Raw data for each of the oestrogens are presented in Fig. 1. Values for the median and interquartile range as well as raw data categorised by autism diagnosis are available in the Supplemental Information (Supplementary Table 2). Oestradiol levels were the most predictive of an autism diagnosis, as revealed by univariate logistic regression (*β* = 0.029, FDR-adjusted *q* = 0.031). The same was found for the levels of oestriol and oestrone, with both hormones being significantly associated to an autism diagnosis using logistic regression (Oestriol: *β* = 0.025, *q* = 0.034; oestrone: *β* = 0.029, FDR-adjusted *q* = 0.031) (Table 2). Oestrone sulphate was also a nominally significant predictor of autism likelihood in the logistic regression model but did not maintain statistical significance following false discovery rate correction (*β* = 0.033, *p* = 0.036, *q* = 0.065) (Table 2).

**Table 1:** Description of Danish Historic Birth Cohort sample

	Control	Autism	
	*n* = 177	*n* = 98	
	Mean ± SD	Mean ± SD	*P* value
Maternal age at birth	33.33 ± 5.15	33.53 ± 5.65	0.775
Paternal age at birth	35.30 ± 6.72	35.85 ± 7.44	0.552
Birth weight (g)	3516.81 ± 659.45	3524.55 ± 679.15	0.928
Gestational week at amniocentesis	14.89 ± 1.91	14.90 ± 1.48	0.953
Storage time (years)	14.90 ± 1.58	14.96 ± 1.69	0.770
APGAR score > 6	97%	96%

**Table 2:** Results of univariate logistic regression for amniotic steroid hormones

	Regression coefficient	Standard error	*z*-value	*p* value	FDR-adjusted *q* value
Oestriol	0.025*	0.010	2.426	0.015	0.034
Oestradiol	0.029*	0.010	2.757	0.006	0.031
Oestrone	0.029*	0.011	2.603	0.009	0.031
Oestrone sulphate	0.033	0.016	2.095	0.036	0.065
Testosterone	0.352	0.304	1.157	0.247	0.247
Androstenedione	0.444	0.270	1.648	0.100	0.128
17-OH Progesterone	0.547	0.271	2.022	0.043	0.065
Progesterone	0.053*	0.021	2.562	0.010	0.031
Cortisol	0.147	0.111	1.332	0.183	0.206

*Statistical significance (*q* < 0.05), following correction via FDR

### Androgens and other steroids

We revisited the previously assayed concentrations of androgens and cortisol [15] in the same subset of samples in which we assayed oestrogens, to understand whether the relationship between oestrogens and autism likelihood was similar to the relationship between androgens and autism likelihood (Table 2, Beeswarm plots of distribution in Supplementary Fig. 2). Of the previously analysed steroid hormones, only progesterone was a significant predictor of autism diagnosis, following univariate logistic regression and correction via FDR in this subset of the cohort (*β* = 0.053, FDR-adjusted *q* = 0.031) (Table 2).

### Comparison

**Fig. 2:**
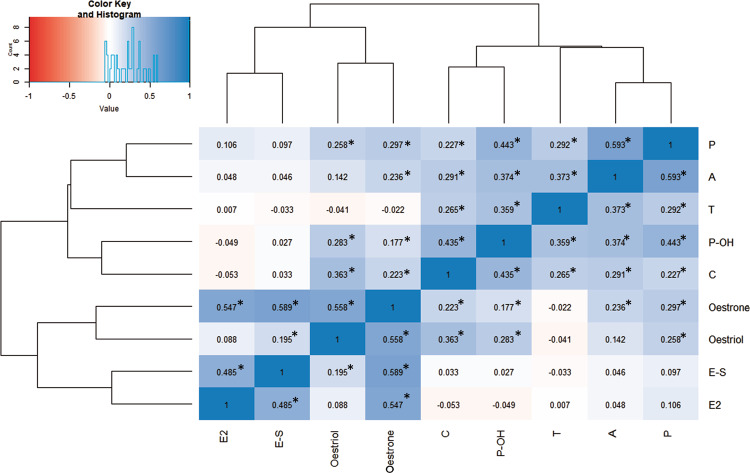
Heatmap and dendrogram of the pairwise correlations between the steroid hormones assayed in amniotic fluid. Asterisk denotes statistical significance (*q* < 0.05), following correction via FDR. P progesterone, A androstenedione, T testosterone; P-OH 17-OH-progesterone, C cortisol, E-S oestrone sulphate, E2 oestradiol

Pairwise correlation analysis (Pearson’s) revealed varying degrees of similarity between the steroid hormones. The concentrations of oestrogens were significantly correlated with one another at *q* < 0.05, adjusted for multiple comparisons via FDR (Fig. 2, Supplementary Table 3). Oestrone and oestriol showed higher correlations to the other steroids than did oestrone sulphate and oestradiol. In comparison, the previously analysed steroid hormones formed a distinct group, with weaker correlations to oestrogens and stronger correlations to each other. Androstenedione and progesterone (Pearson’s *β* = 0.59317, FDR-adjusted *q* < 0.001), oestrone and oestrone sulphate (Pearson’s *β* = 0.589, FDR-adjusted *q* < 0.001) were the pairs that were more closely related. Oestradiol did not correlate with either testosterone or oestriol.

**Fig. 3:**
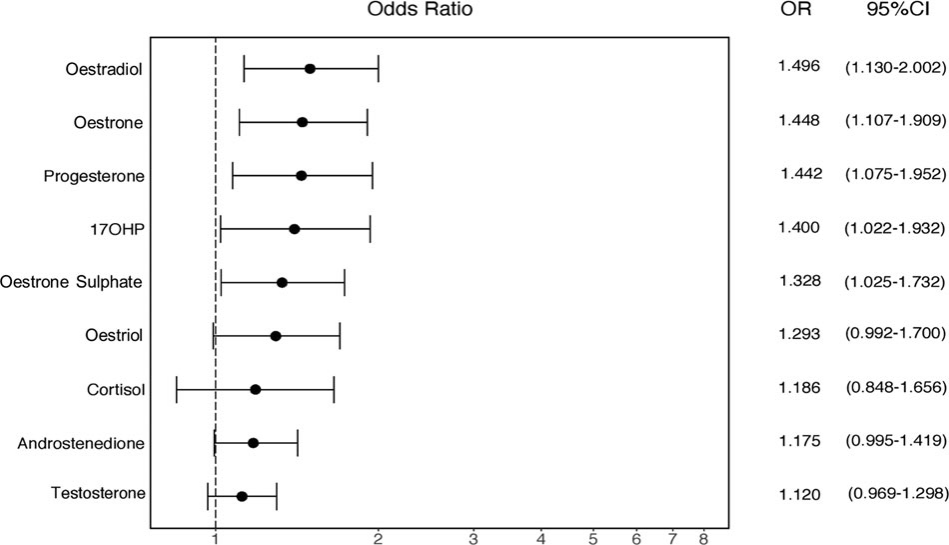
Standardised odds ratios (ORs) for autism diagnosis for all amniotic steroids assayed to date. Each hormone was standardised by its median and interquartile range, so that a one-unit increase in a hormone corresponded to the movement from the 25th to the 75th percentile of its range

We also calculated standardised odds ratios (ORs) for all analysed hormones to determine which hormones had the largest effect on autism likelihood (Fig. 3). Each hormone was standardised by its median and interquartile range, so that a one-unit increase in a hormone corresponded to the movement from the 25th to the 75th percentile of its range. Progesterone and oestradiol had the highest standardised ORs, with a movement from the 25th to 75th percentile of these hormones incurring nearly a 50% increase in autism likelihood. Oestrone and oestrone sulphate also had ORs over 1. In contrast, increases in testosterone or androstenedione levels were not associated with increases in likelihood of diagnosis with statistical confidence.

The aromatising of testosterone (Τ) to oestradiol (Ε2) and of androstenedione (Α) to oestrone (Ε1) were indirectly assessed by calculating their ratios, following log-transformation and subtraction of their raw values. The two ratios were highly correlated with each other (Spearman’s rho = 0.5855, *p* < 0.001) but were not significantly different between autism cases and neurotypical controls (T/E2: Wilcoxon rank-sum *w* = 11186, *p* = 0.1364) (A/E1: Wilcoxon rank-sum *w* = 11032, *p* = 0.2038).

## Discussion

This study reports the first evidence that elevated levels of prenatal amniotic oestradiol, oestriol and oestrone are each associated with autism, with oestradiol levels being the most significant predictor of autism likelihood in univariate logistic regression models. These findings complement earlier observations that elevated steroidogenic activity is associated with autism in the same samples derived from the Danish Historic Birth cohort [15]. We also calculated standardised ORs, in order to directly compare the effect sizes of all amniotic steroids measured to date. We found that oestradiol had the strongest positive effect size on autism likelihood, followed by oestrone, oestriol and progesterone (Fig. 3). This finding appears to contradict an earlier report by Windham et al. [32] that showed that *lower* levels of oestriol in second trimester were modestly associated with a later diagnosis of autism in the offspring. However, our samples correspond to a slightly earlier time point in pregnancy compared to Windham et al. (mean gestational week = 14.9 vs. 17.2 respectively) (see Table 1) [32], which could potentially better capture the steroid surge during the PMW [14]. Furthermore, our samples are of different origin, as Windham et al. assayed maternal serum, rather than foetal amniotic fluid. Steroid hormone levels in maternal serum do not differ relative to the baby’s sex and do not correlate to amniotic levels during the PMW [42]. Therefore, amniotic oestrogens are arguably more relevant to the current research question than are maternal serum oestrogens.

A discrepancy in oestrogen levels between the mother and child could potentially be attributed to the placenta, which acts as an endocrine regulator of the maternal–foetal interface and the main source of oestrogen production for the foetus via the aromatisation of androgens [43]. Several lines of evidence suggest a contributory role for the placenta in the aetiology of autism. First, there is increased placental inflammation in autism [44]. Second, there is atypical placental morphology [45] and increased placental size [46] in cases of autism and at high familial risk respectively. Third, complications related to the placenta (pre-eclampsia [47], hypertensive disorders [48]) are also more frequent in pregnancies that lead to autism. As with autism, placental dysfunction also disproportionately affects males more than females [49].

Given the high pairwise correlations between many of the steroid hormones (Fig. 2, Supplementary Table 3), as well as a lack of difference in aromatisation between cases and controls, our data suggest that an increase in foetal oestrogens is secondary to increased activity along the entirety of the steroidogenic axis in pregnancies that later result in autism [15]. Interestingly, oestradiol was not significantly correlated to testosterone (Pearson’s *β* = 0.007, *p* = 0.9103) despite their proximity in steroidogenesis. This discrepancy may be because oestrogens are also de novo produced by the placenta, in addition to being aromatised from foetal and maternal androgens [43, 50]. Thus, a multi-systems approach is needed in order to clarify the causes of elevated foetal oestrogens in autism.

In the brain, oestrogen-mediated signalling on GABAergic neurons in the hypothalamus is required in order to suppress the steroidogenic axis [51]. Inefficient suppression of this axis in autism could be due to inefficient aromatisation of androgens in the hypothalamus, resistance to oestrogen signalling and/or dysfunction of the GABAergic system. Prenatally, foetal genetics (e.g. due to mutations in aromatase [52] or aromatase activators [53]), pregnancy complications (e.g. placental size [46]), as well as maternal risk factors (e.g. PCOS [18]) could all affect various points in this pathophysiologic pathway. These speculations would require further testing. Specifically for aromatisation, ratios based on circulating hormone levels may not be sufficient to capture tissue-specific activity, since aromatase is differentially regulated by separate promoters in the placenta, the adrenals and the brain [54].

High levels of prenatal oestrogens could dysregulate many aspects of prenatal endocrinology and affect prenatal brain development in areas that are not restricted to sexual differentiation. Several lines of evidence support a wider role of oestradiol as a ‘neurosteroid’ with many regulatory properties [55]. For example, disruption of oestrogen signalling in the developing cerebellum of mice reduces Purkinje cell growth in both males and females, but only reduces social behaviour in male mice, suggesting that the cerebellum may react to oestrogenic disruption in a sexually dimorphic way [56]. In early development, oestradiol decreases GABAergic signalling [57] and mediates its postnatal shift from excitation to inhibition [28, 58]. Oestrogens both increase the number of spines on embryonic primary cortical neurons [55] and induce the recruitment of proteins necessary for excitatory synapse formation, such as neuroligin-1, NMDA subunit GluN1, and post-synaptic density protein 95 (PSD-95) to the spines [59]. Higher levels of prenatal oestrogens might therefore increase the number of excitatory synapses in the cortex, increasing the likelihood for autism, as suggested by the excitatory/inhibitory (E–I) theory of autism [60]. The perceptual phenotype in autism is characterised by reduced GABAergic inhibition, as shown using paradigms such as binocular rivalry [61] and attention to detail [62]. Oestrogen signalling could thus be a significant modulator of neuronal inhibition, particularly during early brain development and the ‘critical period’ of cortical plasticity, which is heavily reliant on the GABAergic system [63].

Although oestradiol (aromatised from testosterone) is the main prenatal masculinising agent in most mammals [24], its role in human sexual differentiation remains unclear. Men with aromatase deficiency have typical development of their urogenital tract [64], but may have cognitive disabilities, lack a growth spurt, and have atypical secondary sexual characteristics such as feminised body proportions in adulthood [65]. Oestrogens may therefore both feminise and masculinise humans, depending on the target tissue and developmental milieu. In autism, cognitive styles and sexually dimorphic neuroanatomy show some masculinised phenotypes [7, 9, 10], but functional connectivity and physical growth show a mixed pattern of masculine and feminine shifts [8, 66]. Prenatally though, and particularly during the masculinisation window, the process of sexual differentiation is understood to be directionally masculine over an anatomically and physiologically female default. The observed high levels of foetal oestrogens could thus be contributing to developmental cognitive differences [10], according to the “extreme male brain” theory of autism.

There was no statistically significant univariate, logistic association between autism and testosterone or androstenedione, which act via the androgen receptor. Mechanisms through which androgenic signalling could increase autism likelihood, which may have been missed in this analysis, include additional androgens or other agonists of the androgen receptor (e.g. neurosteroids like dehydroepiandrosterone [67]), interaction effects between androgens and oestrogens (e.g. coactivation of the androgen receptor by oestradiol [68]), as well as non-linear associations of androgens to autism likelihood. Consequently, androgenic activity may still be an important feature in the development of autism, as suggested by related clinical comorbidities [18, 69] and shown in associations of foetal testosterone to autistic traits in a separate cohort [70].

We could not test whether prenatal oestrogens were associated with autism likelihood in females as there were too few diagnosed women in the HBC in this time window. We plan to test this by expanding the time window. Thus, at present, our findings only generalise to males. Furthermore, comparison of the concentrations of androgens and cortisol to oestrogens is potentially confounded by the fact that the latter were analysed at a later time point and underwent an additional freeze–thaw cycle. We have attempted to minimise any potential sources of confounding by using the same assay methodology with the initial analysis (LC-MS/MS), as well as reassessing for any differences in total storage time in this subset of the original cohort (Table 1).

Another limitation of this study is its reliance on clinical diagnoses from the Danish Central Psychiatric Register, which we could not be independently validated. However, a previous validation study of childhood autism diagnoses in the Danish Central Psychiatric Register found that 94% of diagnoses between 1990 and 1999 in the register were valid using a standardised coding scheme [71]. Similarly, we cannot be certain about the source of amniotic steroids, as they could be of foetal, maternal or placental origin. Foetal plasma and amniotic fluid are in osmotic equilibrium until the foetal skin is keratinised (typically by 25 weeks of gestation) [72]. Therefore, steroid concentrations in amniotic fluid accurately reflect those in foetal circulation.

In conclusion, we have demonstrated that prenatal oestradiol, oestriol and oestrone are elevated in in boys who went on to develop autism. This extends our previous finding of elevated prenatal steroidogenesis in the same cohort and provides further evidence for the prenatal steroid theory of autism [15]. High levels of prenatal oestradiol contribute to a greater degree to autism likelihood than other prenatal sex steroids, including testosterone. We conclude that prenatal oestrogenic excess is a characteristic of autism and may interact with genetic predisposition to affect neurodevelopment.

## Supplementary information

Supplementary Information
