# Supplementary material for: Foetal oestrogens and autism
Source: Mol Psychiatry. 2019 Jul 29;25(11):2970–8. doi: 10.1038/s41380-019-0454-9 (PMC7577840; doi:10.1038/s41380-019-0454-9)
Supplement: Supplementary file 1 — Supplementary Information [file 41380_2019_454_MOESM1_ESM.pdf]

Supplementary Material

Laboratory Methods

Prior to liquid chromatography tandem mass spectroscopy (LC-MS/MS), analytes were extracted from the amniotic fluid samples. Amniotic fluid was diluted with 125 μL of solution containing internal standards for mass spectroscopy. An Oasis WAX μelution plate was used for solid phase extraction. Prior to addition of samples, the plate was washed with 200 μL of methanol (Fischer Scientific, Waltham, MA) and 200 μL of water purified with an in-house MilliQ purifier (EMD Millipore, Billerica, MA). The diluted amniotic fluid samples were added, followed by 200 μL 2% formic acid (Merck, Whitehouse Station, NJ) and 200 μL 10% methanol. The plate was dried under a vacuum for 1 hour, and then analytes were eluted twice with 25 μL of a solution containing 5% ammonium hydroxide (Sigma-Aldrich, St. Louis, MO) 45% acetonitrile (Sigma-Aldrich, St. Louis, MO), and 50% methanol. This extract was further diluted into 50 μL of water.

35 μL of diluted extract was injected into the LC-MS/MS setup. The LC-MS/MS setup consisted of a Waters Acquity Ultra Performance Liquid Chromatography machine with an Acquity sample manager and an Acquity sample organizer, followed by Xevo TQ-S triple quadrupole mass spectrometer equipped with an electron spray ionization probe. Samples were separated on a Poroshell 120 Phenyl Hexyl 2.7μ column (Algilent, Santa Clara, CA) at 50° C. A linear gradient of 0.1% ammonium hydroxide in water (A) to 0.1% of ammonium hydroxide in methanol (B) was used to separate the analytes. The elution gradient followed a pattern of 0.5 min at 80% A and 20% B, 0.5 min at 50% A and 50% B, 3.5 min at 30% A and 70% B, and 5 minutes at 100% B. The quadrupole mass spectrometer was operated in negative mode. Transitions and internal standards are given in Supplementary Table 1. Coefficients of variation (CV) were all 10% or less within the working range of the setup (50 pmol/L to 50 nmol/L).

Supplementary Tables

Supplementary Table 1: Transitions and internal standards used in mass spectroscopy

Analyte	Transition	Internal Standard	Transition
**17β-oestradiol**	271.2/145.1	17β-oestradiol-D3	274.2/145.1
**oestriol**	287.2/171.1		
**oestrone**	269.1/145	Oestrone-D4	273.1/147.1
**oestrone-sulphate**	269.1/145.1	Oestrone-sulphate-D4	273.1/147.1

Supplementary Table 2: Descriptive statistics of oestrogen data. IQR: interquartile range

	**Control**		**Case**	
	n=177		n=98	
	median	IQR	median	IQR
**Oestriol**	2106	[1122, 3091]	2548	[1698, 3399]
**Oestradiol**	181	[32, 330]	245	[70, 421]
**Oestrone**	664	[291, 1036]	877	[431, 1323]
**Oestrone Sulphate**	8895	[4541, 13249]	10605	[5283, 15926]

Supplementary Table 3: Pearson's correlation matrix of amniotic fluid steroid hormones. Asterisk denotes statistical significance (q<0.05), following correction via FDR. T: Testosterone; A: Androstendione; P: Progesterone; P-OH: 17OH-Progesterone; E2: Oestradiol E-S: Oestrone sulphate

	**A**	**P-OH**	**P**	**Cortisol**	**E2**	**Oestriol**	**Oestrone**	**E-S**
**T**	0.37278*	0.359*	0.29175*	0.2646*	0.0068	-0.0414	-0.0220	-0.0327
**A**	1	0.3737*	0.59317*	0.2906*	0.0481	0.1415	0.2360*	0.0463
**P-OH**		1	0.44315*	0.4355*	-0.0488	0.2829*	0.1774*	0.0267
**P**			1	0.2274*	0.10558	0.2584*	0.2974*	0.0969
**Cortisol**				1	-0.0532	0.363*	0.2234*	0.0325
**E2**					1	0.08792	0.5473*	0.4846*
**Oestriol**						1	0.5578*	0.1953*
**Oestrone**							1	0.589*

Supplementary Figures

Supplementary Figure 1. Flowchart illustrating selection of final sample. The initial sample matches the sample from Baron-Cohen *et al.*(2015) prior to the application of data quality selection criteria (i.e., the second to last row in the cohort selection flowchart). We removed cases and controls which had insufficient remaining volume for oestrogen analysis. Finally, we re-applied the data quality selection criteria used in Baron-Cohen *et al.*(2015) (e.g. removal of outliers >99th%, removal of duplicate assays not within 3SD), and obtained a final sample of 98 males with autism and 177 control males.

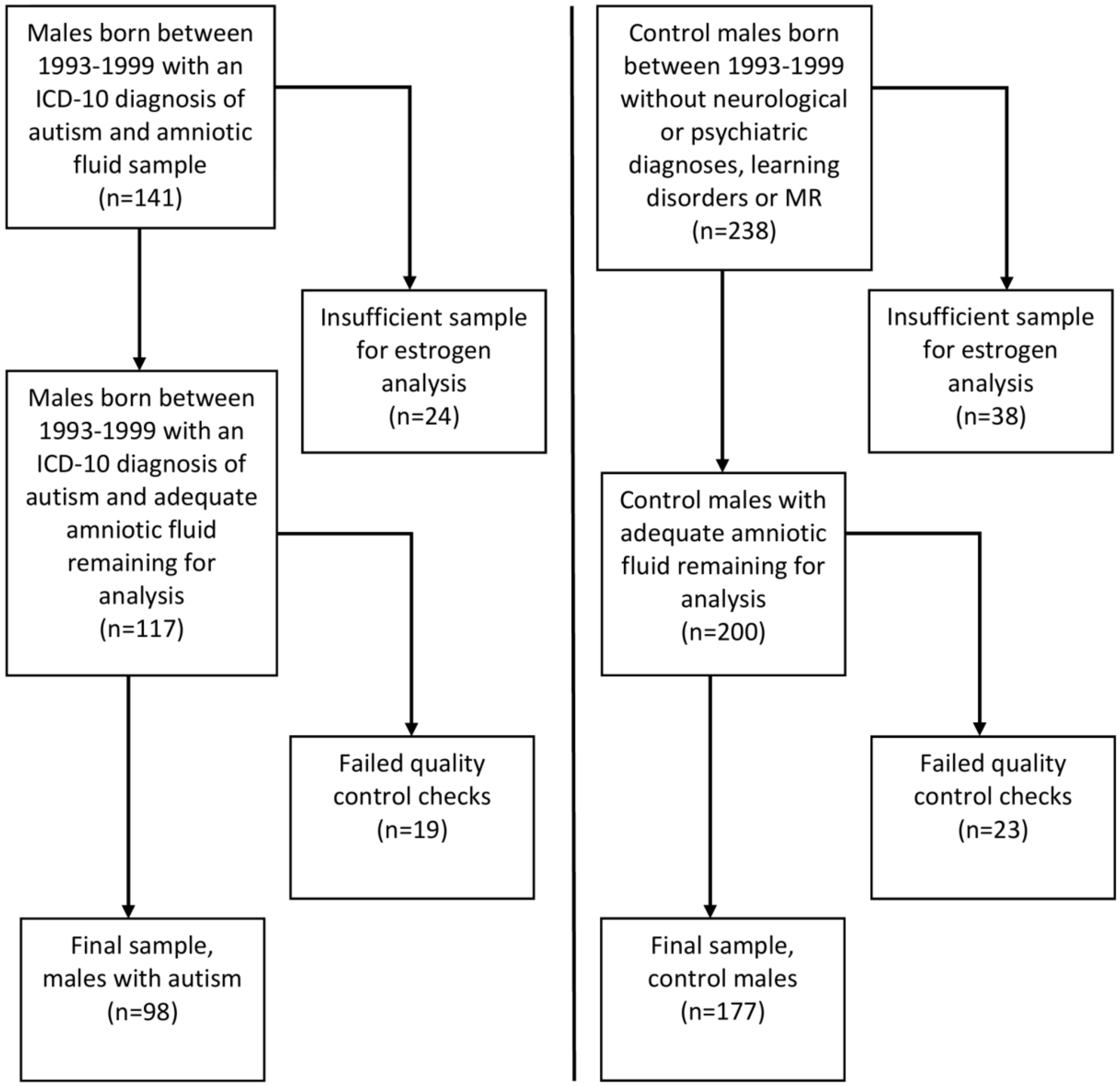

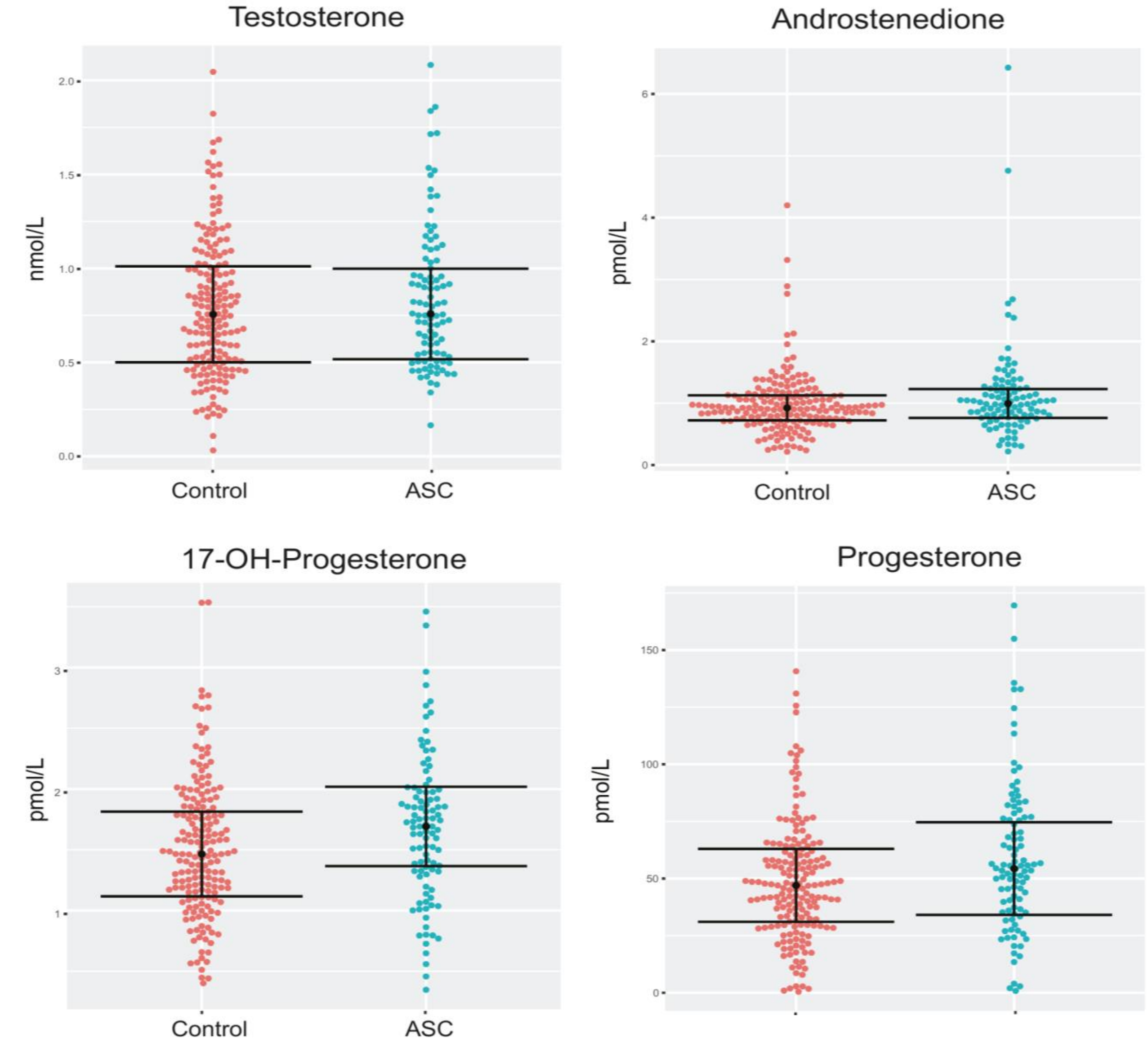

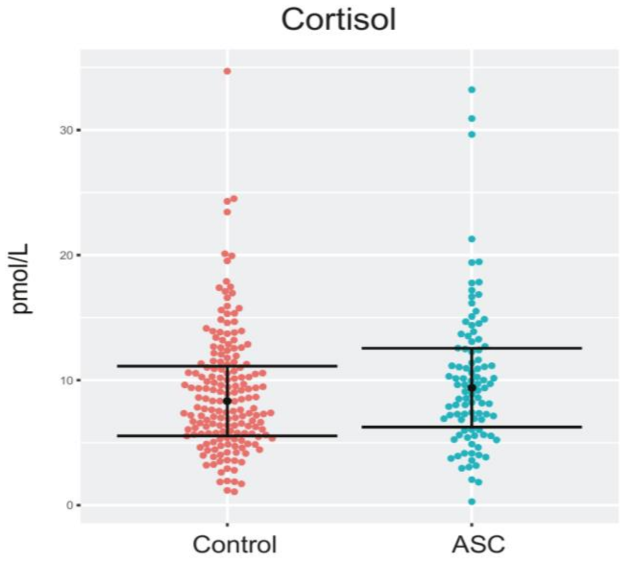

Supplementary Figure 2. Beeswarm plots illustrating the distribution of testosterone, androstenedione, progesterone, cortisol, 17-OH progesterone in the matched sample, as measured by Baron-Cohen *et al.*(2015). Error bars represent the interquartile range, and the black dot represents the median; (*n* CTR = 177, *n* ASC = 98).
